# Supplementary material for: North African humid periods over the past 800,000 years
Source: Nat Commun. 2023 Sep 8;14:5549. doi: 10.1038/s41467-023-41219-4 (PMC10491769; doi:10.1038/s41467-023-41219-4)
Supplement: Supplementary file 1 — Supplementary Information [file 41467_2023_41219_MOESM1_ESM.pdf]

Supplementary Information for:

North African Humid Periods over the past 800000 years

Edward Armstrong[1], Miikka Tallavaara[1], Peter O. Hopcroft[2], Paul J. Valdes[3,4]

1. Department of Geosciences and Geography, University of Helsinki, Finland.
2. School of Geography, Earth and Environmental Sciences, University of Birmingham, UK.
3. School of Geographical Sciences, University of Bristol, UK.
4. Cabot Institute, University of Bristol, UK.

Corresponding author: Edward Armstrong; ORCID: 0000-0001-9561-0159

Contents:

1) Supplementary Figures
   Supplementary Figure 1
   Supplementary Figure 2
   Supplementary Figure 3
   Supplementary Figure 4
   Supplementary Figure 5
   Supplementary Figure 6
   Supplementary Figure 7
   Supplementary Figure 8
   Supplementary Figure 9

2) Model Validation
   Supplementary Figure 10
   Supplementary Figure 11
   Supplementary Figure 12
   Supplementary Figure 13

3) Thermodynamic vs Topographic forcing of the Ice sheet on the NAHPs
   Supplementary Figure 14
   Supplementary Figure 15

1) Supplementary Figures

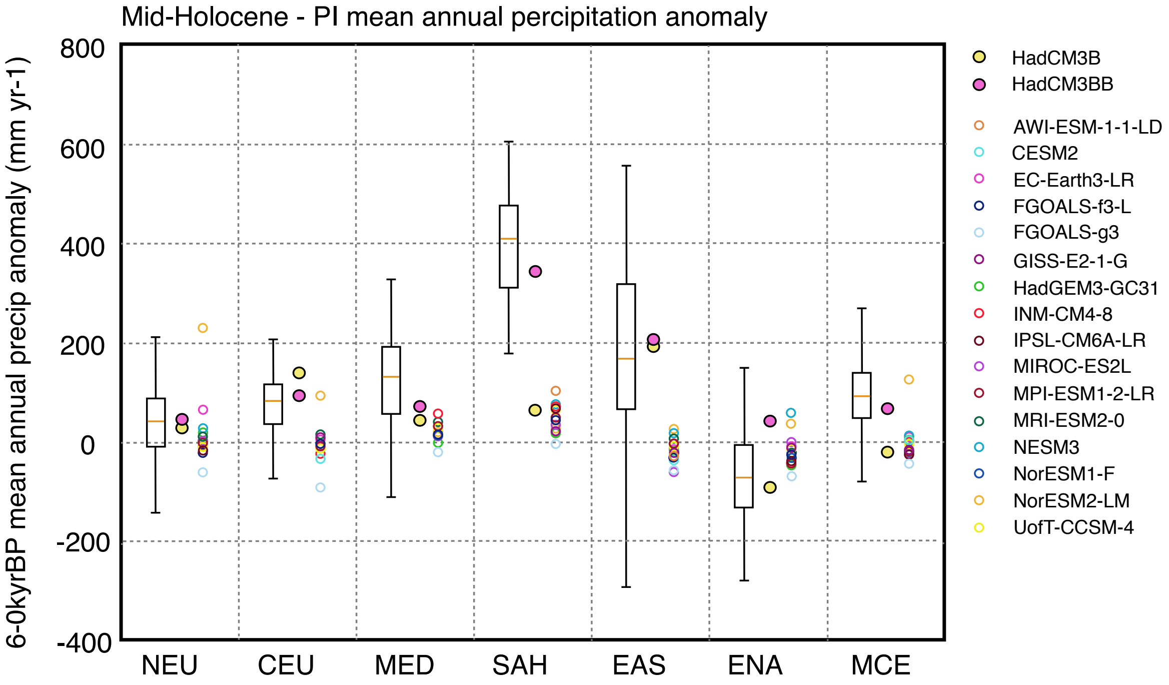

Supplementary Figure 1. Comparison of modelled precipitation changes for a range of climate models against pollen-based reconstructions. Precipitation anomalies are calculated from mid-Holocene (6kyr BP) minus pre-industrial (PI) values and are shown for HadCM3B (filled yellow marker), our convection updated model HadCM3BB-v1.0 (filled purple marker) and a range of other models (coloured markers). Pollen-based reconstructions are shown as box and whiskers [1]. The regions shown follow Figure 8 of Brierley, et al. [2] and are defined as; NEU – Northern Europe, CEU – central Europe, MED – Mediterranean, SAH – Sahara, EAS – Eastern Asia, ENA – Eastern North America, MCE – Mid-continental Eurasia (MCE)[1] (40:60°N, 30:120°E). See Brierley, et al. [2] for more information on the regions and associated references for model data.

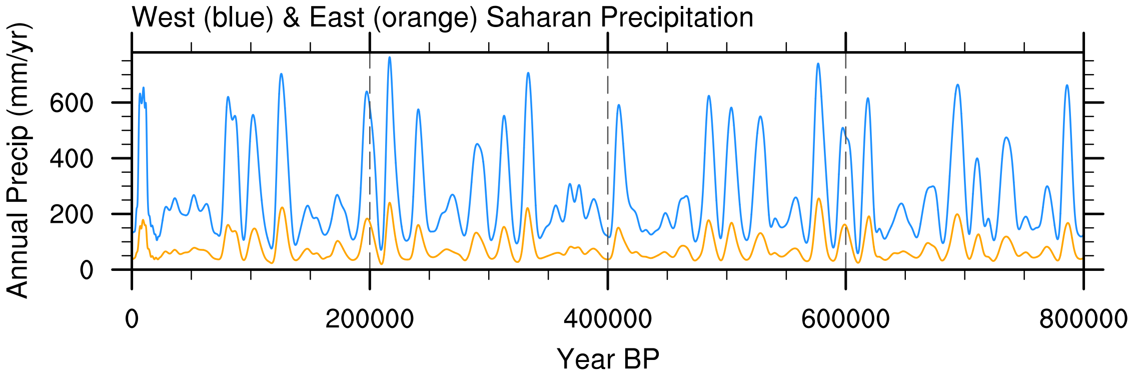

Supplementary Figure 2. Annual precipitation (mm/yr) timeseries for the Western (-15:30°N, 15°W:15°E) and Eastern (-15:30°N, 15°W:50°E) Sahara for 0-800kyr BP.

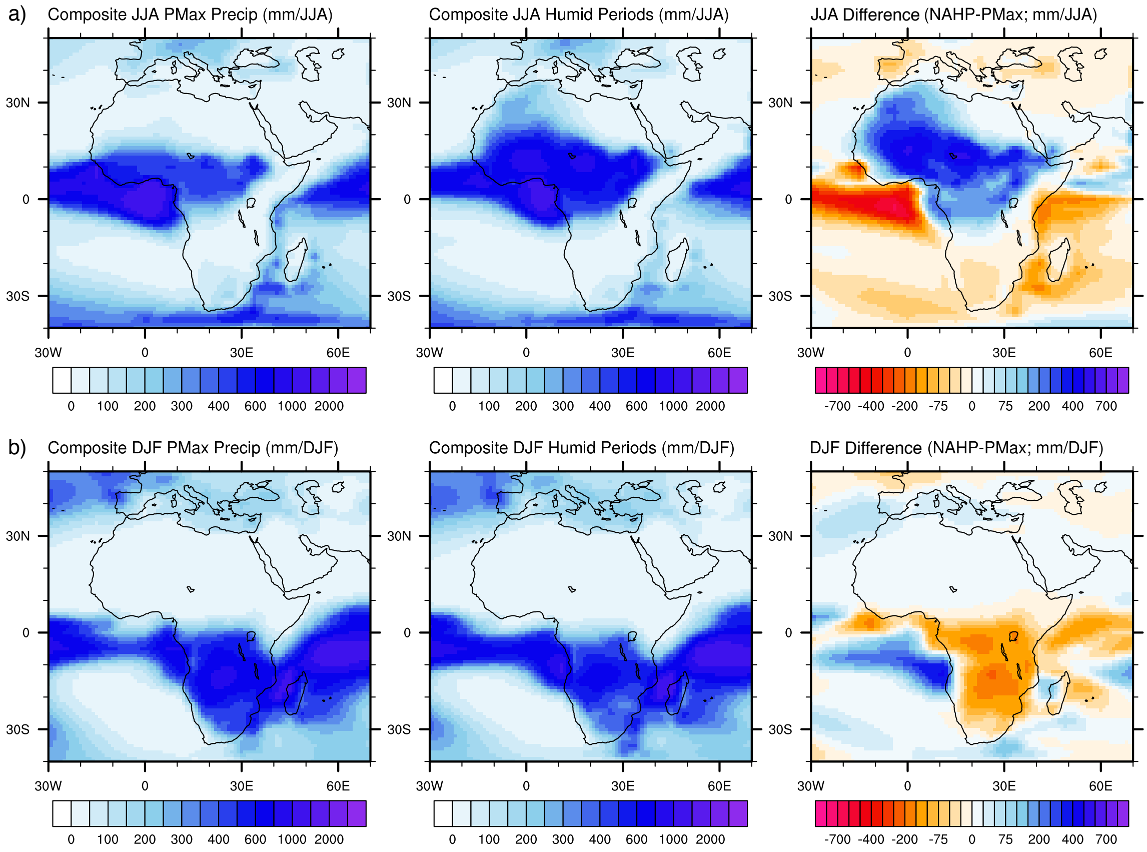

Supplementary Figure 3. Composite seasonal precipitation during periods of precession maxima (PMax), the 20 North African Humid Periods (NAHPs), and the difference between the two (NAHP-PMax). a) Summer (June, July, August; JJA) precipitation (mm/JJA), b) winter (December, January, February; DJF) precipitation (mm/DJF). Only the differences that are considered 95% confident according to a student t-test are shown.

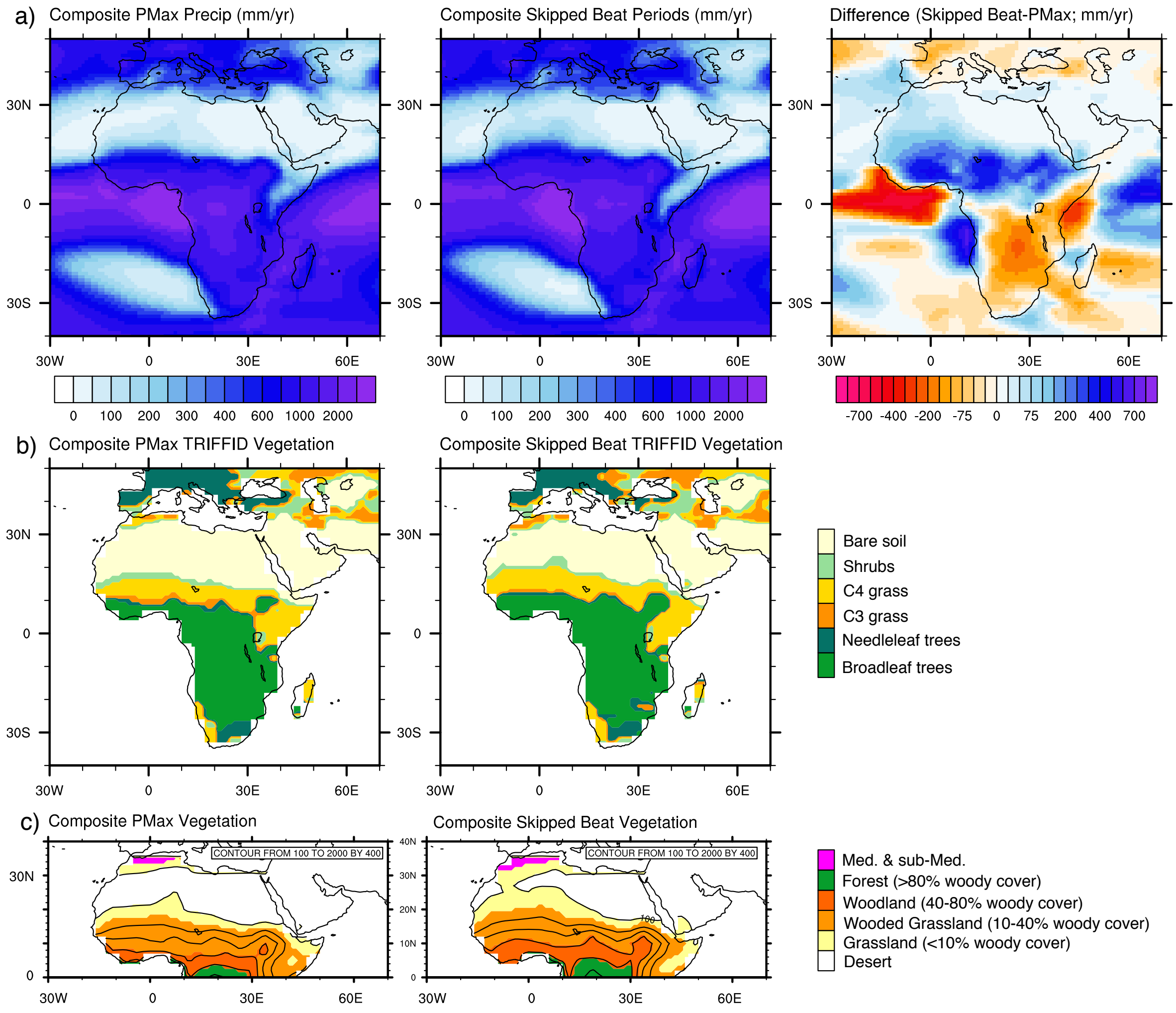

Supplementary Figure 4. Composite precipitation and vegetation during periods of precession maxima (PMax), the 17 skipped beat periods, and the difference between the two (skipped beat-PMax). a) Annual average precipitation (mm/yr), b) vegetation reconstruction from the HadCM3BB-v1.0 dynamic global vegetation model (TRIFFID) showing only the most abundant plant function type (PFT) in each grid square, c) vegetation reconstruction utilising the North African vegetation classification of Larrasoaña, et al. [3] which demonstrates regional vegetation types. Only the differences that are considered 95% confident according to a student t-test are shown.

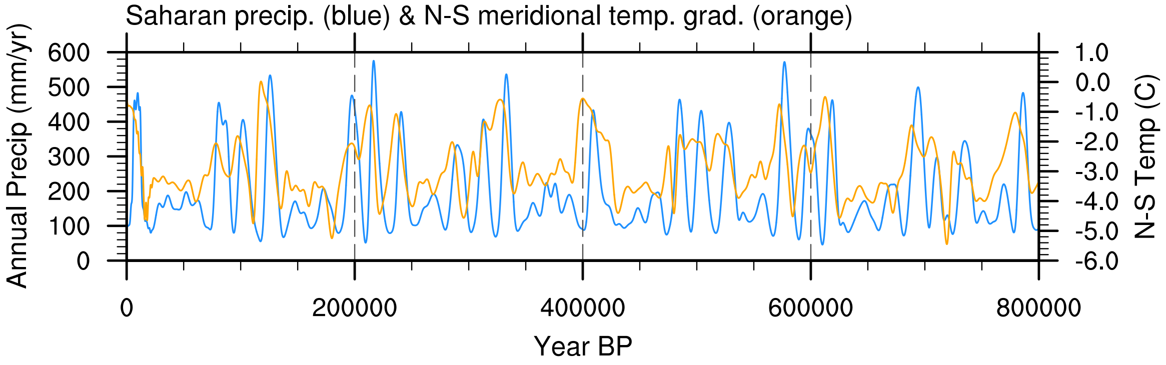

Supplementary Figure 5. Timeseries of modelled Saharan (15:30°N, 15°W:35°E) annual precipitation (mm/yr) and the annual mean global extratropic (poleward of 24°N and 24°S) meridional surface air temperature gradient (°C; North-South).

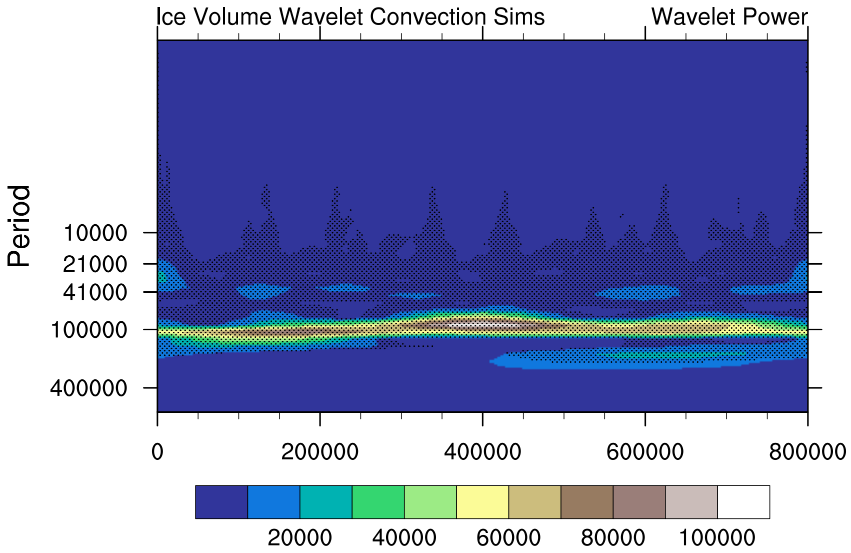

Supplementary Figure 6. Morlet wavelet power spectrum for modelled ice sheet volume. Ice sheet volume is calculated from the ice-sheet model of de Boer, et al. [4], shaded areas highlight power with a 95% confidence level as determined by a red-noise process.

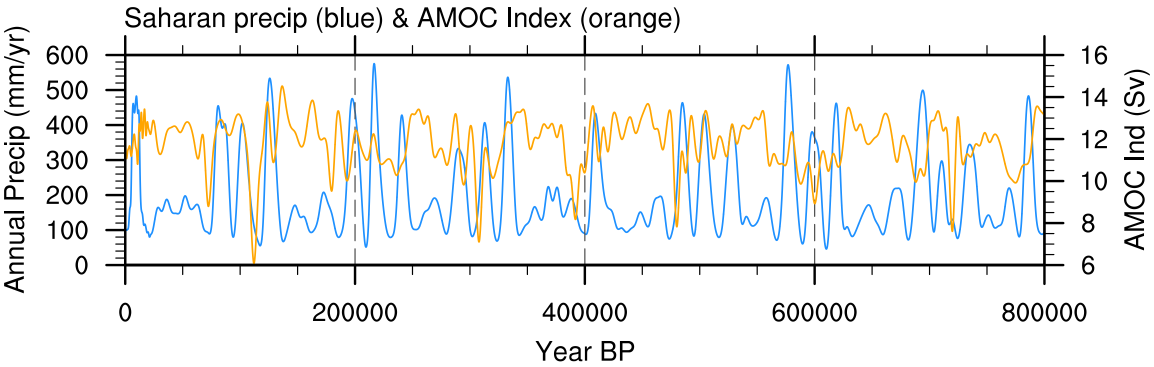

Supplementary Figure 7. Timeseries of modelled Saharan (15:30°N, 15°W:35°E) annual precipitation (mm/yr) and the Atlantic Meridional Overturning Circulation (AMOC) index. The AMOC index is defined as the mean annual strength of the AMOC between 40°N:50°N at 800m water depth.

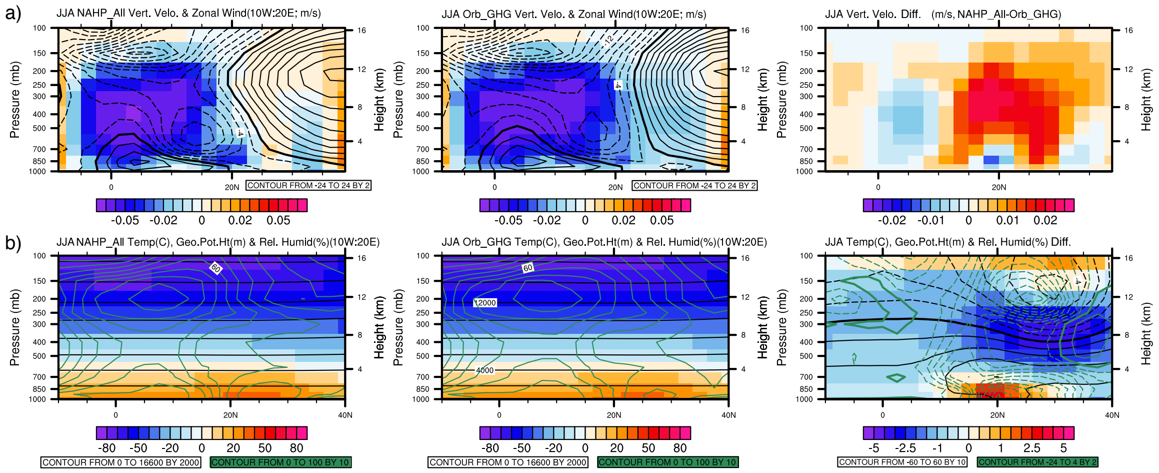

Supplementary Figure 8. Composite latitude/height plots for the Western Sahara (-15:30°N, 10°W:20°E) during the skipped beats, for the NAHP_All experiment, the Orb_GHG experiment, and the difference between the two (NAHP_All-Orb_GHG). The NAHP_All experiment includes all boundary conditions and the Orb_GHG experiment incorporates orbital and greenhouse gas boundary conditions. The difference panels therefore represent the impact of the ice sheet on the climatology. a) Summer (JJA) lagrangian tendency of air pressure (filled colour contours; pa/s) where negative (positive) values represent upward (downward) motion, and zonal wind (black line contours; m/s) where solid/positive (dashed/negative) contours represent westward (eastward) air flow. b) Summer (JJA) temperature (filled colour contours; °C), geopotential height (black line contours; m) and relative humidity (green line contours; %). Solid (dashed) contours represent positive (negative) differences. Only the differences that are considered 95% confident according to a student t-test are shown.

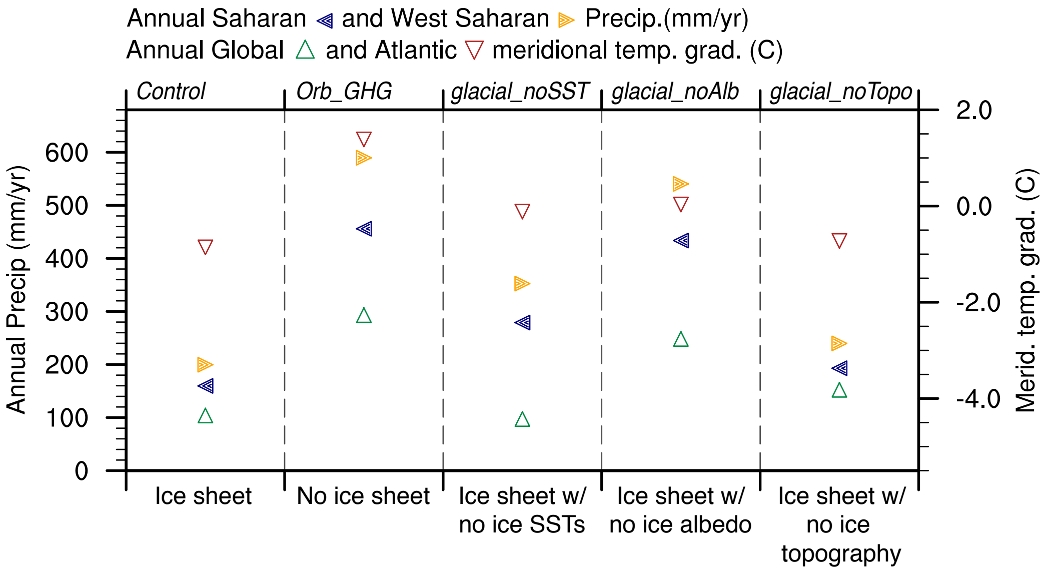

Supplementary Figure 9. Annual mean Saharan (15:30°N, 15°W:35°E) and West Saharan (15:30°N, 15°W:15°E) precipitation, and annual mean global (poleward of 24°N and 24°S) and North Atlantic (poleward of 24°N and 24°S, 70°W:20°E) extratropic meridional temperature gradient (°C; North-South) for the 176kyr BP HadAM3B sensitivity experiments. The associated sensitivity experiments (see Methods) are identified on the Figure and clarify the thermodynamic and topographic impact of the ice sheet on annual average Saharan precipitation and the meridional temperature gradient.

2) Model Validation

A broad validation of the HadCM3B model against a range of observational datasets is provided in section 5 of Valdes, et al. [5]. Their analysis shows that the model accurately reproduces key aspects of the climate system, including surface air temperatures (SATs), sea surface temperatures (SSTs), ocean circulation patters and precipitation. Valdes, et al. [5] also shows that HadCM3B outperforms many of the CMIP5 models particularly with respect to surface air temperatures (see their Figure 2).

The snapshot modelling approach has previously been shown to accurately simulate Quaternary palaeoclimate[6,7], however there are a number of limitations. Firstly, the approach assumes that the climate system is in equilibrium at each time period and that these simulations are a reasonable approximation of the climate at each time period. This assumption has been justified in previous studies[7-9]. Alternative methodologies, such as transient simulations with accelerated forcing, face similar assumptions. The model biases that might affect our simulated climatology are outlined in Valdes, et al. [5], and are comparable to other models. The methodology does not capture millennial scale climate variability, specifically the Dansgaard–Oeschger and Heinrich events which are evident in North Atlantic climate over the Quaternary[10]. In Armstrong, et al. [6], millennial scale variability was added in post processing, by scaling the results of a North Atlantic freshwater hosing experiment with the Greenland Ice core temperature record in order to determine the amplitude and spatial pattern of the DO/HE events. However, the uncertainty associated with this methodology increases with locations further away from Greenland as it utilises the Greenland Ice core record, and so we do not include that methodology here. The model also does not incorporate atmospheric aerosol/dust feedbacks. Despite the uncertainties, our approach is highly efficient as the model simulations can be run simultaneously. A fully continuous simulation would have taken many years to complete on a high-performance computer.

In order to validate the HadCM3BB-v1.0 model, we first compare modelled pre-industrial (0kyr BP, equivalent to the year 1950 without anthropogenic greenhouse gas emissions) precipitation and wind vectors against ERA-5 reanalysis data[11] (Supplementary Figure 10). Although the data is not directly comparable as it represents different time periods, past work has shown that these biases are small compared to the model biases[5]. Note that the model data has been downscaled to 1° resolution via bilinear interpolation. Broadly, the model accurately reproduces synoptic-scale wind patterns, the position of the winter and summer ITCZ, and the spatial pattern/intensity of the West African Monsoon (WAM) over land. In the summer, the model simulates too much precipitation across the Sahara on the order of 0.19mm/day, and very slightly underestimates winter precipitation (-0.04 mm/day). The average annual Saharan precipitation bias is 0.06mm/day equivalent to 21 mm/yr. This positive bias is because the model is prone to drizzle like many GCMs[5], which has a relatively large impact in hyper-arid regions such as the Sahara. This bias will impact the amplitude of any simulated North African Humid periods (NAHPs) in this study, although it is small compared to the magnitude of those events. Outside of the Sahara, both summer and winter precipitation is of comparable magnitude across tropical and subtropical Africa. This is except for precipitation in the region of the Gulf of Guinea which is too high in the model. This may be a result of the stronger simulated regional summer westerlies, which may reflect inaccurate SAT/SSTs in a climatologically complex region which experiences strong upwelling.

However, over land the spatial imprint and intensity the WAM is comparable to observations. The spatial biases in the modelled precipitation, including across the Sahara, are comparable to other CMIP5 models as discussed in Section 5.1.2 of Valdes, et al. [5] (see their Figure 4).

Validating palaeo-model data is challenging due to the lack of reliable observational datasets which are themselves subject to extensive uncertainties and biases. Here we compare model timeseries for SATs, SSTs and precipitation against numerous global proxy datasets covering the past 0-800kyr (Supplementary Figures 11-13) in order to validate in the temporal domain. The associated references are labelled in the Figure captions.

Supplementary Figure 11 shows 12 land-based temperature records including five Greenland ice-core records (b-f)[12-15], two Antarctic ice core records (k, l)[16,17] one of which is a pan-Antarctic records comprised of 5 stacked cores (k)[17], and five records from locations away from the ice sheets (g – j, m)[18-22]. Note that because our modelling approach does not incorporate millennial scale variability, the DO events that dominate the Greenland ice core records are not represented in our simulated dataset. This is particularly evident for the Bolling-Allerod/Younger Dryas interstadial/stadial transition in panels d-f. Despite this, there is generally good agreement in the broad scale patterns in modelled climate with the scale of temperature change in Greenland. However the modelled temperatures are generally too cold during the Holocene and warm beyond. The lack of a Holocene Climatic Optimum in the model has also been shown in a number of other climate models[23], and may reflect biases across the current generation of models used and/or biases with the reconstructions themselves. In Antarctica, there is good agreement with the ice core datasets with regards to absolute temperatures, the timing of the glacial/interglacial cycles, and the scale of temperature change throughout the past 800kyrs. In Africa, the model does well at recreating the observed temperature in Lake Malawi and the Limpopo basin, particularly with regards to the timing of the glacial/interglacial changes. In Lake Tanganyika however the modelled temperatures are apparently too cold. Note however that lake derived temperature reconstructions are subject to a range of uncertainties. Similarly, a LOVECLIM model reconstruction of the Albanian Lake Ohrid indicates that the timing of large-scale temperature changes is well represented, however absolute temperatures are warmer in our model.

Supplementary Figure 12 shows a range of SST records with a wide spatial coverage, albeit generally focused at lower latitudes. In the North Atlantic (b-f)[24-29], the model does generally well at simulating absolute temperatures and the broad scale timing of large-scale temperature changes. However, variability in the observational data is generally greater than that simulated due to the lack of millennial scale variability in the model dataset. There is good agreement with timing of the glacial/interglacial cycles and the extent of temperature change in panels d and f. In the South Atlantic and off Angola (g-i)[30-32], modelled temperatures are too warm, but again the timing of temperature change is consistent. The west coast of Africa is a complex region climatologically due to the presence of strong regional upwelling which may not be properly resolved. In the Indian and Arabian ocean (j-l)[33-35] there is good agreement with modelled absolute temperatures and the patterns of temperature change, albeit with too little variability. There is very good agreement in the SST records in the South China Sea and off Australia (m-o)[33,36,37]. In the Pacific (p-u)[33,38-44] modelled temperature are generally too warm, however the patterns of temperature change are broadly consistent with the observations.

Validating palaeo-precipitation is more challenging that SATs and SSTs due to the lack of comparable observational datasets. Palaeo-precipitation proxies such as lake levels, geochemical composition of sediments, and vegetation composition (pollen, $\delta^{13}C$) do not easily translate into quantitative precipitation estimates, but instead track relative changes in the precipitation or moisture availability. These proxies may also be influenced by additional factors other than precipitation which can hamper direct comparison. Furthermore, precipitation is problematic due to its significant spatial heterogeneity; therefore, observational records may demonstrate localised trends which may not be captured by a coarse scale model. For example, precipitation is sensitive to orography and the position of the coastlines, which is particularly subject to model resolution and may introduce biases. This may influence direct comparison in regions such as the Eastern African rift valley and around the complex coastline of the Mediterranean.

Supplementary Figure 13 shows a collection of Mediterranean and African proxies for moisture availability. In the Mediterranean and North Africa (b-c)[21,45], the observation timeseries show Quercus (deciduous Oak) abundance (b) from Lake Ohrid in Albania and Ba/Al which tracks sapropel intervals from Eastern Mediterranean sediments (c). Both records indicate clear precession and eccentricity forcing, with periods of enhanced moisture availability at precession minima during interglacials and suppression during glacials. In both panels (b-c), the timing of enhanced moisture coincides with the NAHPs identified in our model timeseries (Figure 1), and both observations lend support to the conclusions of this study. There is a good correlation between the modelled precipitation in the Nile River Basin and high Mediterranean Ba/Al intervals. In the case of Lake Ohrid, comparison with model data is complicated because this region is not resolved in the model due to low resolution of the coastline, therefore the model timeseries shown in panel b is from a region to the North of Lake Ohrid. This is likely why there is disagreement with the model data, such as the lack of an eccentricity signal. However, precession timing is broadly consistent for both model and observations.

In the Western Sahara (d-e)[46,47], modelled precipitation shows good agreement with patterns of dust deposition in marine sediments (d) and humidity change inferred from grain-size analyses of siliciclastic marine sediments (e). There is consistent precession timing in both the model and observations, with strong peaks in the observations during the interglacials and broadly weaker peaks during glacial precession minima. In tropical West Africa (f)[48], pollen abundance from a sediment core off Liberia is of poor temporal resolution but demonstrates enhanced peaks at around 100kyr BP and 200kyr BP which coincides with interglacials, indicative of increased precipitation during these periods and increased aridity during glacials.

Lake Bosomtwe woody cover data (g-h) has recently been published with two different age models[49,50] yielding different temporal patterns. Both records show variability on a precession timescale, but the different age models appear to create significant differences in the larger scale patterning between the two versions of the proxy. This highlights the problems of model-proxy comparisons. In panel g, the interglacial timing corresponds more accurately with the model data, and there is also generally better correlation with the strong precession variability demonstrated by the model in this region.

In central Africa, terrigenous mineral composition of sediment cores from the Zaire fan (j-k)[51,52] shows very good correlation with the model data, although precession is more dominant in the observation record in panel j. Both records and model demonstrate precession and obliquity orbital signals, and generally enhanced river run-off and moisture availability during precession minima and interglacials.

Eastern Africa, which includes the East African Rift valley (i, l-o)[19,53-56], is a complex climatological region influenced by highly variable topography and a number of atmospheric systems including the walker circulation, the East African monsoon and El-Nino. A record of Ca/Ti from Lake Tana in Ethiopia is indicative of lake level (i), and the authors state[53] that it shows evidence for a more stable wet environment during the Holocene and the last interglacial between ~132–70 ka BP, although this is not clear in the observations. The record shows some agreement with our model data, but it does not demonstrate the same dominant precession forcing.

A potassium-based aridity record from the Chew Bahir basin in southern Ethiopia (l) shows good correlation with the model data (note that this data has been obtained from the study of Kaboth-Bahr, et al. [57] as the original data is not available). The observations show clear dominant precession peaks modulated by eccentricity, the timing of which agree well with the model data. A 400kyr eccentricity signal is also apparent in both the model and observation data, and a trend towards increasing aridity since the last interglacial. A pollen record from Lake Magadi in Kenya (m) supposedly shows aridification of the region over the past 500kyr[55], however the temporal resolution of the record is poor prior to 250kyr BP. This long-term aridification is not apparent in the model data, and the proxy does not show a strong trend either. Overall there is generally poor correlation between the model and Lake Magadi data. This is also apparent for the three precession minima peaks during the last interglacial between 70-120kyr, which are too closely grouped together in the observation dataset compared to the model and orbital data.

A leaf wax carbon isotopic record from the Kenyan Koora Basin (n) shows precession forcing strongly modulated by eccentricity, with particularly high amplitude variability during the penultimate interglacial between 180-270kyr BP. Although there is some disagreement between the model and the timing of some of the precession peaks, there is very good model-data agreement with the eccentricity signal which indicates increased/decreased moisture availability during interglacials/glacials, and the enhanced impact of precession during the penultimate interglacial. A lake level record from Lake Malawi (o) shows, according to the authors[19], evidence for 100kyr eccentricity with moist interglacial and dry glacials. However, the lake level data does not show precession forcing which is in contrast to the model data. However, the model does capture some of the megadroughts identified in the Lake Malawi data of Lyons, et al. [58].

In Southern Africa (p-q)[59,60], precession variability is antiphased with that in Northern and Central Africa, with enhanced precipitation during precession maxima (enhanced austral summer insolation). A leaf-wax δD record from a marine core off Namibia shows dominant precession forcing which correlates very well with model variability. Both the model and data indicate aridification in the region since the last glacial maximum. A Fe/Ca record from the Limpopo basin in South East Africa (q) show clear precession peaks that correlate well with

our model data. There is also good agreement between the model and observations of stronger precession forcing during eccentricity maxima. However the model does not indicate increasing aridity between 800 to 600kyr as implied by the authors[59], but this trend does not appear to be strong in the proxy data either.

In summary, the HadCM3BB-v1.0 model accurately simulates present-day precipitation and wind patterns over Africa with biases comparable to other CMIP5 models, including the position of the ITCZ and WAM. Temporally, the model and the snapshot modelling approach that we utilise is shown to produce a climatology that is comparable to proxy datasets for SATs, SSTs and precipitation, in terms of absolute values and patterns of change over the past 800kyr. This model/data agreement lends strong support to the overall approach that we have employed.

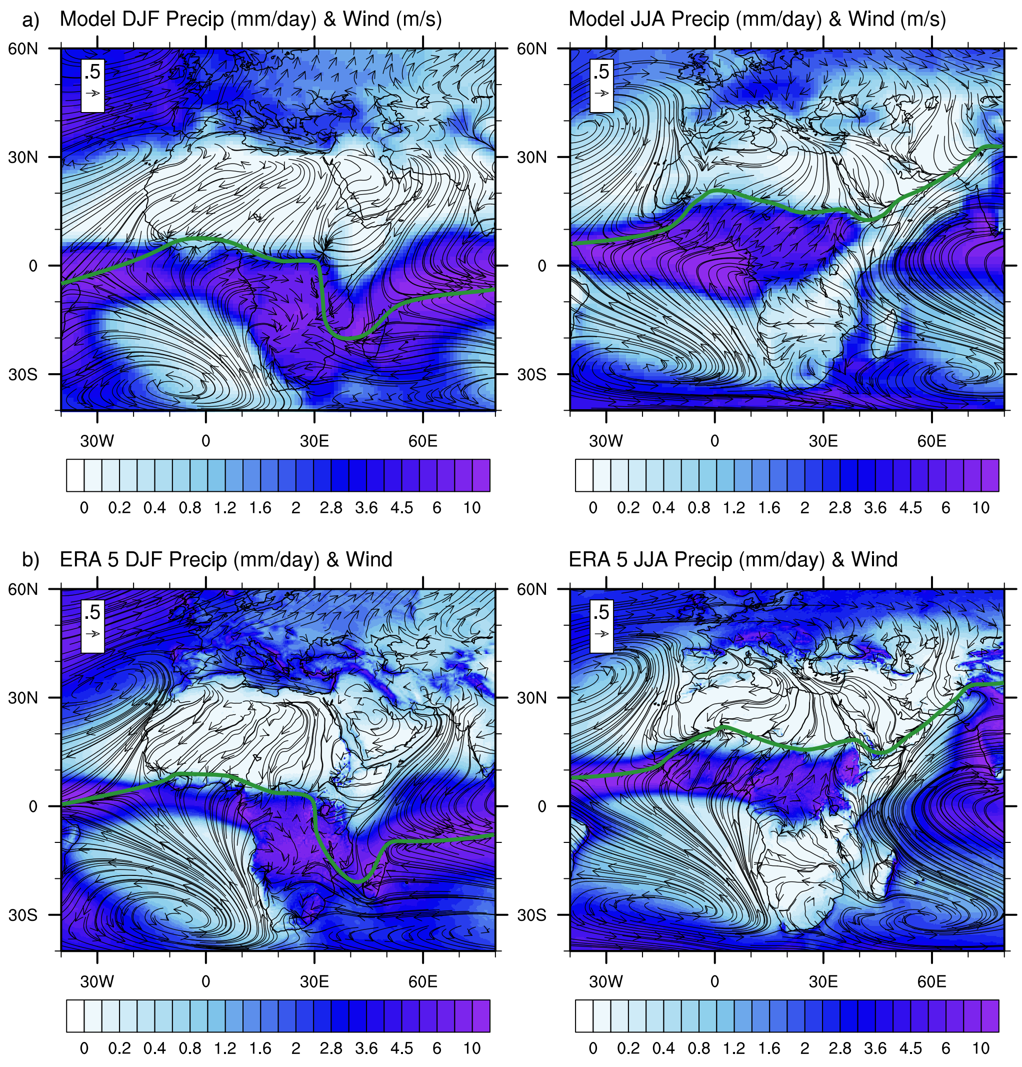

Supplementary Figure 10. A comparison of pre-industrial HadCM3BB-v1.0 model output with ERA-5 reanalysis data. a) Winter and summer pre-industrial (0 kyr BP) HadCM3BB-v1.0 output downscaled to 1° resolution for precipitation, wind and the position of the Intertropical Convergence Zone (ITCZ). b) Same as above but for ERA-5 reanalysis data[11] averaged over the period 1951-2020. The ITCZ is defined as where the northern and southern trade winds converge.

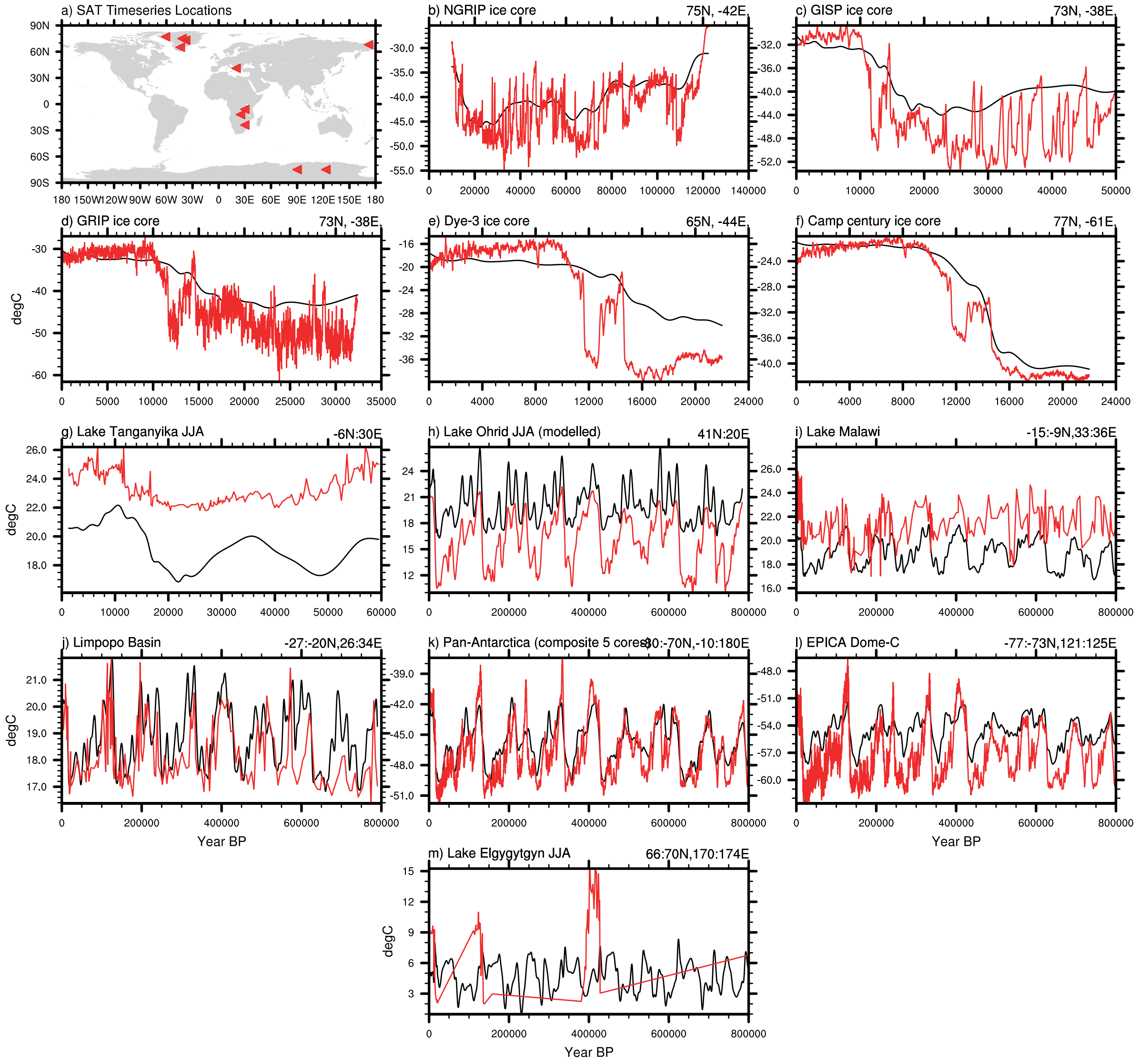

Supplementary Figure 11. Validation timeseries plots for surface air temperatures (°C). Simulated temperatures (black) are shown with a range of observational datasets (red), the locations of which are shown in panel a, and the specific lat/lon position for each dataset is given above each panel. Millennial scale variability (i.e. the Dansgaard–Oeschger and Heinrich events) are absent from the model data due to the snapshot approach that we use. Note the changes in the x-axis. The associated references are: b[14], c[13], d[15], e[12], f[12], g[20], h[21], i[19], j[18], k[17], l[16], m[22].

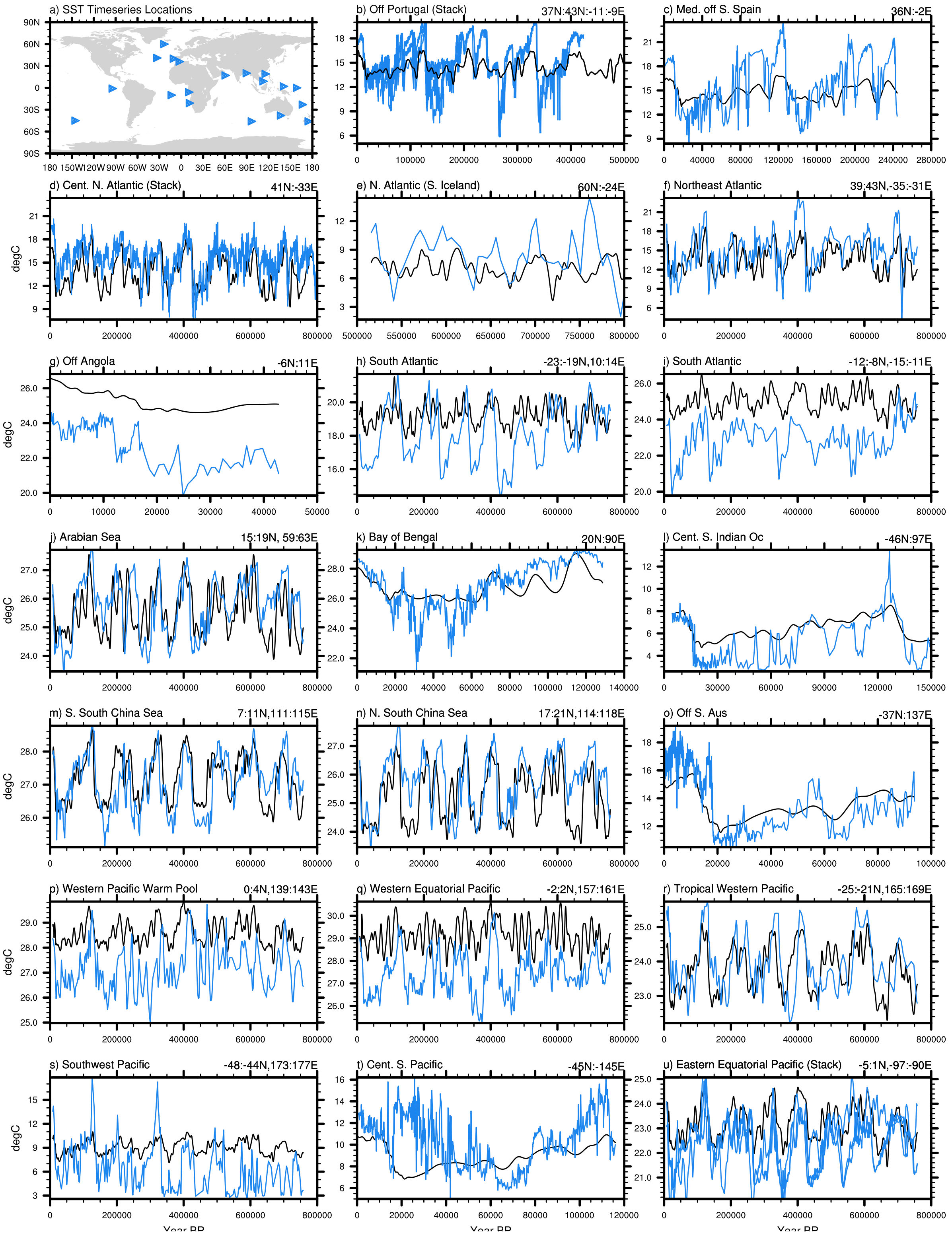

Supplementary Figure 12. Validation timeseries plots for sea surface temperatures (°C). The same as Supplementary Figure 11 but for sea surface temperatures (°C), with observation locations shown in panel a. The associated references are: b[25,26,28], c[26], d[24,27], e[24], f[29], g[32], h[30], i[31], j[33], k[34], l[35], m[37], n[33], o[36] ,p[39], q[42], r[43], s[44], t[40], u[33,38,41].

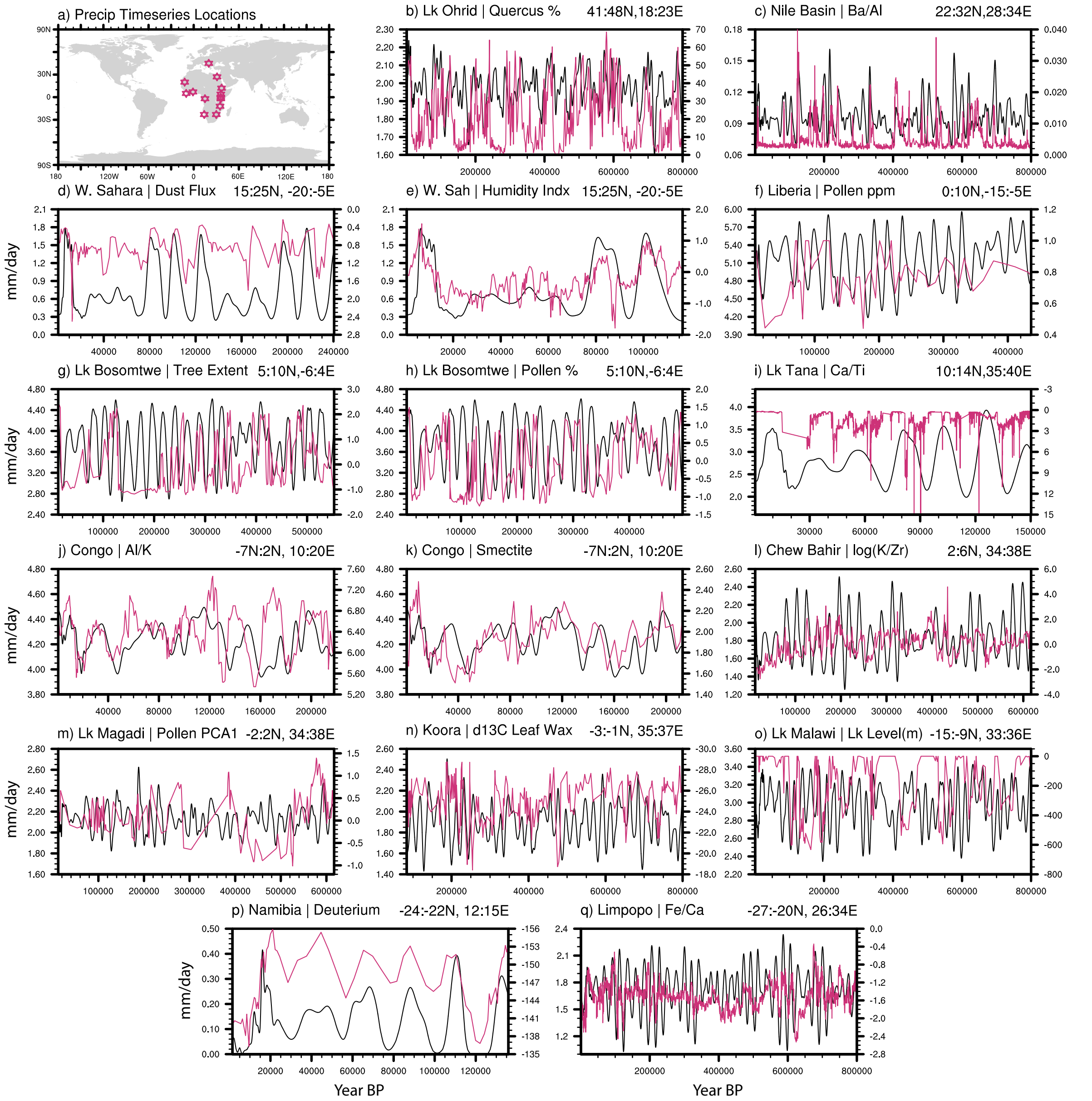

Supplementary Figure 13. Validation timeseries plots for precipitation (mm/day). The same as Supplementary Figures 11 and 12 but for precipitation, with observation locations shown in panel a. All left y-axis labels are for model data in mm/day and right axis units are outlined in the panel label. The associated references are : b[21], c[45], d[46], e[47], f[48], g[50], h[49], l[53], j[52], k[51], l[56] ,m[55], n[54], o[19], p[60], q[59].

3) Thermodynamic vs Topographic forcing of the Ice sheet on the NAHPs

Our results show that during glacial periods, extensive ice sheets act to suppress the NAHPs during periods of precession minima. The impact of the ice sheets may be both a thermodynamic and/or topographic forcing. The thermodynamic mechanism reflects the increase in the surface albedo due to the ice sheet, which reduces surface air temperatures (SATs) and/or sea surface temperatures (SSTs). This may alter the meridional temperature gradient, the position of the ITCZ and reduce the strength and northward propagation of the African monsoon systems during precession minima. The topographic effect reflects the impact of the ice sheet elevation on large-scale wind patterns, which might impact ITCZ/monsoon positioning and/or intensity.

In order to identify whether the thermodynamic or topographic mechanism is dominant, we performed additional sensitivity experiments as outlined in the Methods. We focus on one time period (176kyr BP) due to computation limitations, the mechanism that we identify at this time is assumed to be the case for each of the skipped beats. We have taken the glacial simulation with the ice sheet; we have then successively altered specific glacial forcings associated with the ice sheet – SSTs, ice sheet albedo, and ice sheet topography (see methods).

Supplementary Figure 9 shows the average annual Saharan precipitation for the Control HadAM3BB-v1.0 simulation (i.e. the atmosphere only version of the model with all glacial forcings), the 176kyr HadCM3BB-v1.0 Orb_GHG simulation (i.e. no ice sheet forcings), the glacial_noSST simulation (i.e. glacial forcings except for non-glacial SSTs prescribed from the 176kyr Orb_GHG experiment), the glacial_noAlb simulation (i.e. glacial forcings except for pre-industrial albedo), and the glacial_noTopo simulation (i.e. glacial forcings except for pre-industrial topography). The global and Atlantic meridional temperature gradient for each simulation is also shown. The results show that the inclusion of the ice sheet results in a decrease in Saharan precipitation at 176kyr BP by approximately 65% as indicated by the difference between Orb_GHG and Control. When removing the influence of glacial SSTs (glacial_noSST), this precipitation decrease is approximately 39%. When removing the influence of glacial albedo (glacial_noAlb), precipitation decreases by only approximately 5%. When removing the influence of glacial topography (glacial_noTopo), precipitation decreases by 58%. It is evident therefore that it is the impact of glacial albedo change and its influence on the atmosphere that is the dominant driver of reduced precipitation during glacial periods. There is a smaller but still significant contribution from SST changes. The topographic impact is minimal in comparison. Note that the impact of these three forcings is non-linear, and when combined do not produce the overall ice sheet anomaly.

Supplementary Figure 14 shows the spatial pattern of African summer precipitation for the 176kyr NAHP_All and Orb_GHG snapshot simulations (panel a) and the HadAM3BB-v1.0 sensitivity experiments (panels b-e). The anomalies represent the change in precipitation due to the inclusion of the ice sheet (a-b), or the inclusion of the specific ice sheet forcings (SST, albedo, topography; panels c-e). We show the NAHP_All climatology and anomaly with Orb_GHG (panel a) in order to confirm that the Control HadAM3BB simulation is producing a very similar climatology to the coupled climate model (panel b). The spatial plots confirm the findings in Supplementary Figure 9, showing that the inclusion of glacial albedo and its impact

on the atmosphere has a dominant impact in reducing glacial precipitation, with a secondary impact from glacial sea-surface temperatures. The impact of glacial SSTs primarily reduces precipitation between 10-20°N whereas the impact of albedo change is primarily between 10-35°N.

Latitude/height plots (Supplementary Figure 8, Supplementary Figure 15a-b) indicate that during glacial precession minima periods, the ice sheets cool the atmosphere, weaken the West African Monsoon (WAM), strengthen (weaken) the African Easterly Jet (Tropical Easterly Jet), and cause the sub-tropical westerly jet (STWJ) to descend which inhibits precipitation. This is similar to the mechanism of precipitation suppression during precession maxima (Figure 2b).

The contribution of glacial SSTs (Supplementary Figure 15c-d) primarily weakens the WAM and alters the position of the African/Tropical Easterly jets, which decreases uplift and precipitation between 10-20°N (Supplementary Figure 15c). There is little impact on the dynamics of the sub-tropical westerly jet and only a small cooling impact on atmospheric temperatures (Supplementary Figure 15d). The broad impact of the ice sheets is to cool SSTs, specifically in the North Atlantic, sub-tropical gyre and the Mediterranean. This may influence the land-sea thermal contrast around West Africa and the Gulf of Guinea, which consequently influences the strength of the WAM. However the direct mechanism by which this occurs, and which oceanic regions are responsible for this, is beyond the scope of this study. Glacial SSTs also cause a more negative Atlantic meridional temperature gradient, although there is little impact on the global meridional gradient (Supplementary Figure 9). This may contribute in shifting the ITCZ to a more southerly position, as has been shown in other studies[61,62].

The atmospheric anomalies due to the impact of glacial albedo (Supplementary Figure 15e-f) is very similar to that shown by the overall addition of the ice sheets (Supplementary Figure 15a-b). Albedo change weakens the WAM, alters the position and strength of the African/Tropical Easterly jets, and causes a northward shift, strengthening, and descending sub-tropical westerly jet. This inhibits uplift and convective rainfall between 10-35°N. This is due to the widespread cooling of the troposphere north of 10°N. This also results in a more negative global and Atlantic meridional temperature gradient (Supplementary Figure 9), which may act to shift the ITCZ to a more southerly position during glacials[61,62]. These results indicate that it is albedo change and the resultant tropospheric cooling which is responsible for much of the suppression of Saharan precipitation during glacials (Supplementary Figure 15f).

In summary, ice sheets suppress the NAHPs during period of precession minima primarily due to the thermodynamic impact of albedo change. This cools the atmosphere which reduces the meridional temperature gradient, weakens the WAM and alters the dynamics of the sub-tropical westerly jet which inhibits uplift. In addition, glacial SSTs suppress precession-induced strengthening of the WAM.

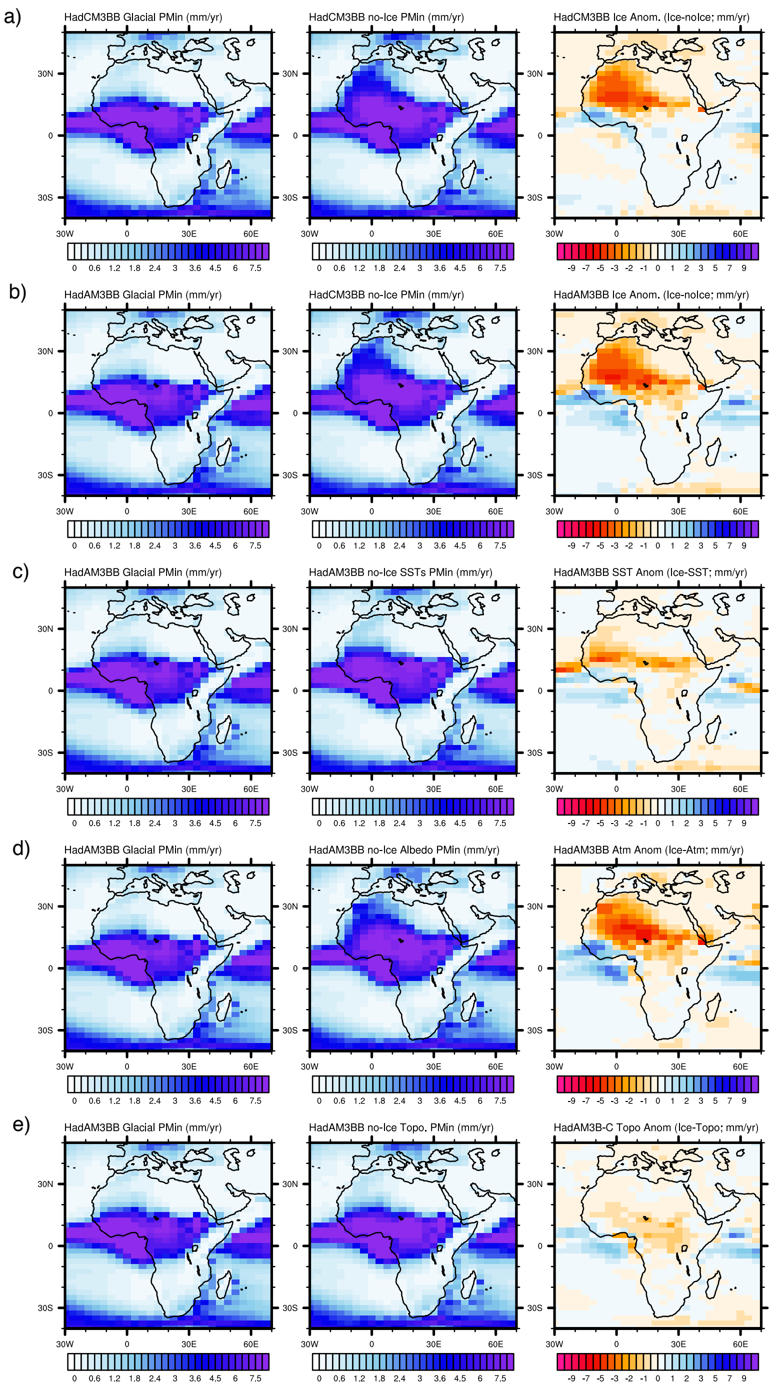

Supplementary Figure 14. Thermodynamic and topographic Impacts of ice sheet addition on summer (JJA) precipitation at 176kyr BP. a) Summer (JJA) precipitation (mm/day) for HadCM3BB-v1.0 NAHP_All, HadCM3BB-v1.0 Orb_GHG, and the anomaly which represents the change in precipitation due to the addition of the ice sheet. b) same as above but utilising the Control HadAM3BB simulation to confirm the precipitation anomaly for the atmosphere only model, c) summer precipitation for Control, glacial_noSST and the anomaly representing change in precipitation due to addition of glacial SSTs, d) summer precipitation for control, glacial_noAlb and the anomaly representing change in precipitation due to addition of glacial albedo and its effect on the atmosphere, and e) summer precipitation for control, glacial_noTopo and the anomaly representing change in precipitation due to addition of glacial topography.

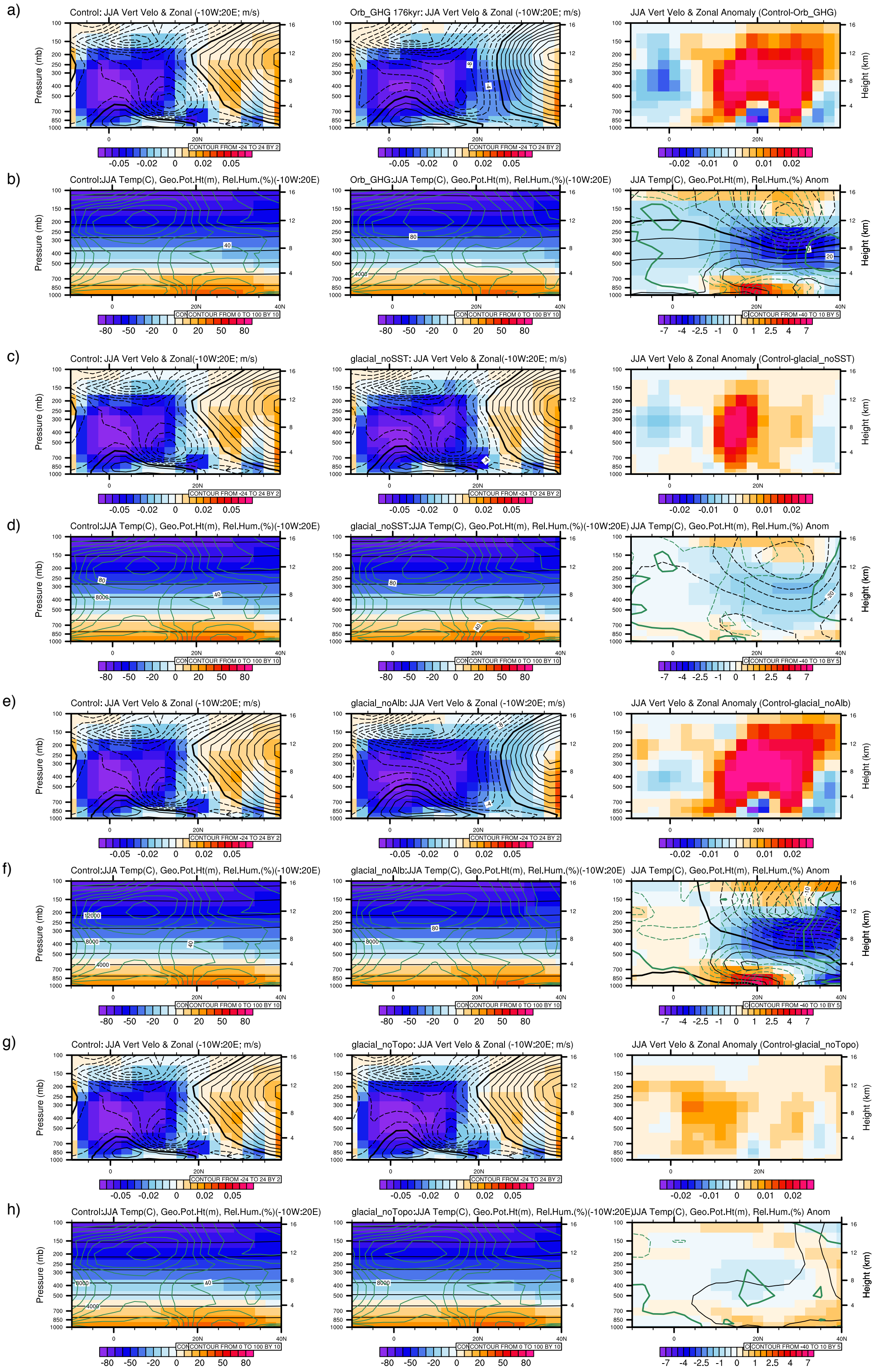

Supplementary Figure 15. West Saharan summer (JJA) latitude/height climatologies at 176kyr BP for the Control simulation, the thermodynamic/topographic sensitivity experiments, and

the anomaly between the two (sensitivity experiment - Control). The region is identified in pink in Figure 2, and the specific experiments are Orb_GHG (panels a-b), glacial_noSST (panels c-d), glacial_noAlb (panels e-f), and glacial_noTopo (panels g-h). These are defined in each panel label. Panels a, c, e, g) the lagrangian tendency of air pressure for (filled colour contours; pa/s) where negative (positive) values represent upward (downward) motion, and zonal wind (black line contours; m/s) where solid/positive (dashed/negative) contours represent westward (eastward) air flow. The major components of the West African Monsoon (WAM) are identified in Figure 2b. Panels b, d, f, h) temperature (filled colour contours; °C), geopotential height (black line contours; m) and relative humidity (green line contours; %). Solid (dashed) contours represent positive (negative) anomalies. Anomalies are 95% confident according to a student t-test.

Supplementary References

1 Bartlein, P. J. *et al.* Pollen-based continental climate reconstructions at 6 and 21 ka: a global synthesis. *Climate Dynamics* **37**, 775-802, doi:10.1007/s00382-010-0904-1 (2011).

2 Brierley, C. M. *et al.* Large-scale features and evaluation of the PMIP4-CMIP6 midHolocene simulations. *Climate of the Past* **16**, 1847-1872, doi:10.5194/cp-16-1847-2020 (2020).

3 Larrasoaña, J. C., Roberts, A. P. & Rohling, E. J. Dynamics of Green Sahara Periods and Their Role in Hominin Evolution. *PLoS ONE* **8**, e76514, doi:10.1371/journal.pone.0076514 (2013).

4 de Boer, B., Lourens, L. J. & van de Wal, R. S. W. Persistent 400,000-year variability of Antarctic ice volume and the carbon cycle is revealed throughout the Plio-Pleistocene. *Nature Communications* **5**, doi:10.1038/ncomms3999 (2014).

5 Valdes, P. J. *et al.* The BRIDGE HadCM3 family of climate models: HadCM3@Bristol v1.0. *Geoscientific Model Development* **10**, 3715-3743, doi:10.5194/gmd-10-3715-2017 (2017).

6 Armstrong, E., Hopcroft, P. O. & Valdes, P. J. A simulated Northern Hemisphere terrestrial climate dataset for the past 60,000 years. *Scientific Data* **6**, doi:10.1038/s41597-019-0277-1 (2019).

7 Singarayer, J. S. & Valdes, P. J. High-latitude climate sensitivity to ice-sheet forcing over the last 120 kyr. *Quaternary Science Reviews* **29**, 43-55, doi:10.1016/j.quascirev.2009.10.011 (2010).

8 Armstrong, E., Valdes, P., House, J. & Singarayer, J. The Role of CO2 and Dynamic Vegetation on the Impact of Temperate Land-Use Change in the HadCM3 Coupled Climate Model. *Earth Interactions* **20**, 1-20, doi:10.1175/ei-d-15-0036.1 (2016).

9 Davies-Barnard, T., Ridgwell, A., Singarayer, J. & Valdes, P. Quantifying the influence of the terrestrial biosphere on glacial-interglacial climate dynamics. *Climate of the Past* **13**, 1381-1401, doi:10.5194/cp-13-1381-2017 (2017).

10 Dansgaard, W. *et al.* EVIDENCE FOR GENERAL INSTABILITY OF PAST CLIMATE FROM A 250-KYR ICE-CORE RECORD. *Nature* **364**, 218-220, doi:10.1038/364218a0 (1993).

11 Hersbach, H. *et al.* The ERA5 global reanalysis. *Quarterly Journal of the Royal Meteorological Society* **146**, 1999-2049, doi:10.1002/qj.3803 (2020).

12 Buizert, C. *et al.* Greenland-Wide Seasonal Temperatures During the Last Deglaciation. *Geophysical Research Letters* **45**, 1905-1914, doi:10.1002/2017gl075601 (2018).

13 Cuffey, K. M. & Clow, G. D. Temperature, accumulation, and ice sheet elevation in central Greenland through the last deglacial transition. *Journal of Geophysical Research-Oceans* **102**, 26383-26396, doi:10.1029/96jc03981 (1997).

14 Kindler, P. *et al.* Temperature reconstruction from 10 to 120 kyr b2k from the NGRIP ice core. *Climate of the Past* **10**, 887-902, doi:10.5194/cp-10-887-2014 (2014).

15 Rasmussen, S. O. *et al.* A new Greenland ice core chronology for the last glacial termination. *Journal of Geophysical Research-Atmospheres* **111**, doi:10.1029/2005jd006079 (2006).

16 Jouzel, J. *et al.* Orbital and millennial Antarctic climate variability over the past 800,000 years. *Science* **317**, 793-796, doi:10.1126/science.1141038 (2007).

17 Parrenin, F. *et al.* Synchronous Change of Atmospheric CO2 and Antarctic Temperature During the Last Deglacial Warming. *Science* **339**, 1060-1063, doi:10.1126/science.1226368 (2013).
18 Chevalier, M., Chase, B. M., Quick, L. J., Dupont, L. M. & Johnson, T. C. Temperature change in subtropical southeastern Africa during the past 790,000 yr. *Geology* **49**, 71-75, doi:10.1130/g47841.1 (2021).
19 Johnson, T. C. *et al.* A progressively wetter climate in southern East Africa over the past 1.3 million years. *Nature* **537**, 220-+, doi:10.1038/nature19065 (2016).
20 Tierney, J. E. *et al.* Northern hemisphere controls on tropical southeast African climate during the past 60,000 years. *Science* **322**, 252-255, doi:10.1126/science.1160485 (2008).
21 Wagner, B. *et al.* Mediterranean winter rainfall in phase with African monsoons during the past 1.36 million years. *Nature* **573**, 256-+, doi:10.1038/s41586-019-1529-0 (2019).
22 Melles, M. *et al.* 2.8 Million Years of Arctic Climate Change from Lake El'gygytgyn, NE Russia. *Science* **337**, 315-320, doi:10.1126/science.1222135 (2012).
23 Liu, Z. Y. *et al.* The Holocene temperature conundrum. *Proceedings of the National Academy of Sciences of the United States of America* **111**, E3501-E3505, doi:10.1073/pnas.1407229111 (2014).
24 Bahr, A. *et al.* Oceanic heat pulses fueling moisture transport towards continental Europe across the mid-Pleistocene transition. *Quaternary Science Reviews* **179**, 48-58, doi:10.1016/j.quascirev.2017.11.009 (2018).
25 Hodell, D. *et al.* Response of Iberian Margin sediments to orbital and suborbital forcing over the past 420 ka. *Paleoceanography* **28**, doi:10.1002/palo.20017 (2013).
26 Martrat, B. *et al.* Four climate cycles of recurring deep and surface water destabilizations on the Iberian margin. *Science* **317**, 502-507, doi:10.1126/science.1139994 (2007).
27 Naafs, B. D. A., Hefter, J. & Stein, R. Millennial-scale ice rafting events and Hudson Strait Heinrich(-like) Events during the late Pliocene and Pleistocene: a review. *Quaternary Science Reviews* **80**, 1-28, doi:10.1016/j.quascirev.2013.08.014 (2013).
28 Rodrigues, T., Voelker, A. H. L., Grimalt, J. O., Abrantes, F. & Naughton, F. Iberian Margin sea surface temperature during MIS 15 to 9 (580-300 ka): Glacial suborbital variability versus interglacial stability. *Paleoceanography* **26**, doi:10.1029/2010pa001927 (2011).
29 Ruddiman, W. F., Raymo, M. E., Martinson, D. G., Clement, B. M. & Backman, J. PLEISTOCENE EVOLUTION: NORTHERN HEMISPHERE ICE SHEETS AND NORTH ATLANTIC OCEAN. *Paleoceanography* **4**, 353-412, doi:10.1029/PA004i004p00353 (1989).
30 Etourneau, J., Schneider, R., Blanz, T. & Martinez, P. Intensification of the Walker and Hadley atmospheric circulations during the Pliocene-Pleistocene climate transition. *Earth and Planetary Science Letters* **297**, 103-110, doi:10.1016/j.epsl.2010.06.010 (2010).
31 McIntyre, A., Ruddiman, W. F., Karlin, K. & Mix, A. C. SURFACE WATER RESPONSE OF THE EQUATORIAL ATLANTIC OCEAN TO ORBITAL FORCING. *Paleoceanography* **4**, 19-55, doi:10.1029/PA004i001p00019 (1989).

32 Rampen, S. W. *et al.* Long chain 1,13-and 1,15-diols as a potential proxy for palaeotemperature reconstruction. *Geochimica Et Cosmochimica Acta* **84**, 204-216, doi:10.1016/j.gca.2012.01.024 (2012).
33 Herbert, T. D., Peterson, L. C., Lawrence, K. T. & Liu, Z. H. Tropical Ocean Temperatures Over the Past 3.5 Million Years. *Science* **328**, 1530-1534, doi:10.1126/science.1185435 (2010).
34 Lauterbach, S. *et al.* An similar to 130 kyr Record of Surface Water Temperature and delta O-18 From the Northern Bay of Bengal: Investigating the Linkage Between Heinrich Events and Weak Monsoon Intervals in Asia. *Paleoceanography and Paleoclimatology* **35**, doi:10.1029/2019pa003646 (2020).
35 Rickaby, R. E. M. & Elderfield, H. Planktonic foraminiferal Cd/Ca: Paleonutrients or paleotemperature? *Paleoceanography* **14**, 293-303, doi:10.1029/1999pa900007 (1999).
36 De Deckker, P. *et al.* Climatic evolution in the Australian region over the last 94 ka - spanning human occupancy -, and unveiling the Last Glacial Maximum. *Quaternary Science Reviews* **249**, doi:10.1016/j.quascirev.2020.106593 (2020).
37 Li, L. *et al.* A 4-Ma record of thermal evolution in the tropical western Pacific and its implications on climate change. *Earth and Planetary Science Letters* **309**, 10-20, doi:10.1016/j.epsl.2011.04.016 (2011).
38 Barth, A. M., Clark, P. U., Bill, N. S., He, F. & Pisias, N. G. Climate evolution across the Mid-Brunhes Transition. *Climate of the Past* **14**, 2071-2087, doi:10.5194/cp-14-2071-2018 (2018).
39 de Garidel-Thoron, T., Rosenthal, Y., Bassinot, F. & Beaufort, L. Stable sea surface temperatures in the western Pacific warm pool over the past 1.75 million years. *Nature* **433**, 294-298, doi:10.1038/nature03189 (2005).
40 Harada, N. *et al.* Rapid fluctuation of alkenone temperature in the southwestern Okhotsk Sea during the past 120 ky. *Global and Planetary Change* **53**, 29-46, doi:10.1016/j.gloplacha.2006.01.010 (2006).
41 Liu, Z. H. & Herbert, T. D. High-latitude influence on the eastern equatorial Pacific climate in the early Pleistocene epoch. *Nature* **427**, 720-723, doi:10.1038/nature02338 (2004).
42 Medina-Elizalde, M. & Lea, D. W. The mid-Pleistocene transition in the tropical Pacific. *Science* **310**, 1009-1012, doi:10.1126/science.1115933 (2005).
43 Russon, T. *et al.* Inter-hemispheric asymmetry in the early Pleistocene Pacific warm pool. *Geophysical Research Letters* **37**, doi:10.1029/2010gl043191 (2010).
44 Schaefer, G. *et al.* Planktic foraminiferal and sea surface temperature record during the last 1 Myr across the Subtropical Front, Southwest Pacific. *Marine Micropaleontology* **54**, 191-212, doi:10.1016/j.marmicro.2004.12.001 (2005).
45 Grant, K. M. *et al.* Organic carbon burial in Mediterranean sapropels intensified during Green Sahara Periods since 3.2 Myr ago. *Communications Earth & Environment* **3**, doi:10.1038/s43247-021-00339-9 (2022).
46 Skonieczny, C. *et al.* Monsoon-driven Saharan dust variability over the past 240,000 years. *Sci Adv* **5**, doi:10.1126/sciadv.aav1887 (2019).
47 Tjallingii, R. *et al.* Coherent high- and low-latitude control of the northwest African hydrological balance. *Nature Geoscience* **1**, 670-675, doi:10.1038/ngeo289 (2008).

48 Jahns, S., Huls, M. & Sarnthein, M. Vegetation and climate history of west equatorial Africa based on a marine pollen record off Liberia (site GIK 16776) covering the last 400,000 years. *Review of Palaeobotany and Palynology* **102**, 277-288 (1998).
49 Gosling, W. D. *et al.* A stronger role for long-term moisture change than for CO2 in determining tropical woody vegetation change. *Science* **376**, 653-+, doi:10.1126/science.abg4618 (2022).
50 Miller, C. S., Gosling, W. D., Kemp, D. B., Coe, A. L. & Gilmour, I. Drivers of ecosystem and climate change in tropical West Africa over the past similar to 540 000 years. *Journal of Quaternary Science* **31**, 671-677, doi:10.1002/jqs.2893 (2016).
51 Gingele, F. X., Muller, P. M. & Schneider, R. R. Orbital forcing of freshwater input in the Zaire Fan area - clay mineral evidence from the last 200 kyr. *Palaeogeography Palaeoclimatology Palaeoecology* **138**, 17-26, doi:10.1016/s0031-0182(97)00121-1 (1998).
52 Schneider, R. R., Price, B., Muller, P. J., Kroon, D. & Alexander, I. Monsoon related variations in Zaire (Congo) sediment load and influence of fluvial silicate supply on marine productivity in the east equatorial Atlantic during the last 200,000 years. *Paleoceanography* **12**, 463-481, doi:10.1029/96pa03640 (1997).
53 Lamb, H. F. *et al.* 150,000-year palaeoclimate record from northern Ethiopia supports early, multiple dispersals of modern humans from Africa. *Scientific Reports* **8**, doi:10.1038/s41598-018-19601-w (2018).
54 Lupien, R. L. *et al.* Eastern African environmental variation and its role in the evolution and cultural change of Homo over the last 1 million years. *Journal of Human Evolution* **157**, doi:10.1016/j.jhevol.2021.103028 (2021).
55 Owen, R. B. *et al.* Progressive aridification in East Africa over the last half million years and implications for human evolution. *Proceedings of the National Academy of Sciences of the United States of America* **115**, 11174-11179, doi:10.1073/pnas.1801357115 (2018).
56 Trauth, M. H. *et al.* Recurring types of variability and transitions in the similar to 620 kyr record of climate change from the Chew Bahir basin, southern Ethiopia. *Quaternary Science Reviews* **266**, doi:10.1016/j.quascirev.2020.106777 (2021).
57 Kaboth-Bahr, S. *et al.* Paleo-ENSO influence on African environments and early modern humans. *Proceedings of the National Academy of Sciences* **118**, e2018277118, doi:doi:10.1073/pnas.2018277118 (2021).
58 Lyons, R. P. *et al.* Continuous 1.3-million-year record of East African hydroclimate, and implications for patterns of evolution and biodiversity. *Proceedings of the National Academy of Sciences of the United States of America* **112**, 15568-15573, doi:10.1073/pnas.1512864112 (2015).
59 Caley, T. *et al.* A two-million-year-long hydroclimatic context for hominin evolution in southeastern Africa. *Nature* **560**, 76-+, doi:10.1038/s41586-018-0309-6 (2018).
60 Collins, J. A., Schefuss, E., Govin, A., Mulitza, S. & Tiedemann, R. Insolation and glacial-interglacial control on southwestern African hydroclimate over the past 140 000 years. *Earth and Planetary Science Letters* **398**, 1-10, doi:10.1016/j.epsl.2014.04.034 (2014).
61 Biasutti, M. Rainfall trends in the African Sahel: Characteristics, processes, and causes. *Wiley Interdisciplinary Reviews-Climate Change* **10**, doi:10.1002/wcc.591 (2019).

62 Mohino, E., Janicot, S. & Bader, J. Sahel rainfall and decadal to multi-decadal sea surface temperature variability. *Climate Dynamics* **37**, 419-440, doi:10.1007/s00382-010-0867-2 (2011).
